# Isolating Action Prediction from Action Integration in the Perception of Social Interactions

**DOI:** 10.3390/brainsci12040432

**Published:** 2022-03-24

**Authors:** Ana Pesquita, Ulysses Bernardet, Bethany E. Richards, Ole Jensen, Kimron Shapiro

**Affiliations:** 1 Centre for Human Brain Health, School of Psychology, University of Birmingham, Birmingham B15 2TT, UK; ber893@student.bham.ac.uk (B.E.R.); o.jensen@bham.ac.uk (O.J.); k.l.shapiro@bham.ac.uk (K.S.); 2 Aston Institute of Urban Technology and the Environment (ASTUTE), Aston University, Birmingham B4 7ET, UK; u.bernardet@aston.ac.uk

**Keywords:** social interaction, action integration, action prediction, autistic traits

## Abstract

Previous research suggests that predictive mechanisms are essential in perceiving social interactions. However, these studies did not isolate *action prediction* (a priori expectations about how partners in an interaction react to one another) from *action integration* (a posteriori processing of both partner’s actions). This study investigated action prediction during social interactions while controlling for integration confounds. Twenty participants viewed 3D animations depicting an action–reaction interaction between two actors. At the start of each action–reaction interaction, one actor performs a social action. Immediately after, instead of presenting the other actor’s reaction, a black screen covers the animation for a short time (occlusion duration) until a still frame depicting a precise moment of the reaction is shown (reaction frame). The moment shown in the reaction frame is either temporally aligned with the occlusion duration or deviates by 150 ms or 300 ms. Fifty percent of the action–reaction trials were semantically congruent, and the remaining were incongruent, e.g., one actor offers to shake hands, and the other reciprocally shakes their hand (congruent action–reaction) versus one actor offers to shake hands, and the other leans down (incongruent action–reaction). Participants made fast congruency judgments. We hypothesized that judging the congruency of action–reaction sequences is aided by temporal predictions. The findings supported this hypothesis; linear speed-accuracy scores showed that congruency judgments were facilitated by a temporally aligned occlusion duration, and reaction frames compared to 300 ms deviations, thus suggesting that observers internally simulate the temporal unfolding of an observed social interction. Furthermore, we explored the link between participants with higher autistic traits and their sensitivity to temporal deviations. Overall, the study offers new evidence of prediction mechanisms underpinning the perception of social interactions in isolation from action integration confounds.

## 1. Introduction

Imagine that you are washing the dishes with your partner after hosting a party: one washes, the other rinses. A guest who observes you from afar exclaims, ‘Oops…careful,’ just as you are about to fail to grab one of the slippery dishes that your partner hands over. In this scenario, action prediction allows you and your partner to choose suitable complementary actions while washing the dishes together [1,2]. Previous literature suggests that besides supporting the execution of social interactions, prediction also facilitates their observation [3,4,5,6]. Hence, in the previous scenario, the guest’s perception of the interaction is likewise supported by predictive representations. Individuals observing interactions from a third-person perspective create expectations about what might happen next in the interaction even though they are not directly engaged. This study focuses on reassessing the contribution of predictive mechanisms to the perception of social interactions. We note that previous research on this topic has not fully disambiguated between *action prediction* and *action integration*. Following this observation, we designed the present study to experimentally isolate a priori expectations about action–reaction interactions (*action prediction*) from the a posteriori integration of both partners’ actions (*action integration*).

**Figure 1:**
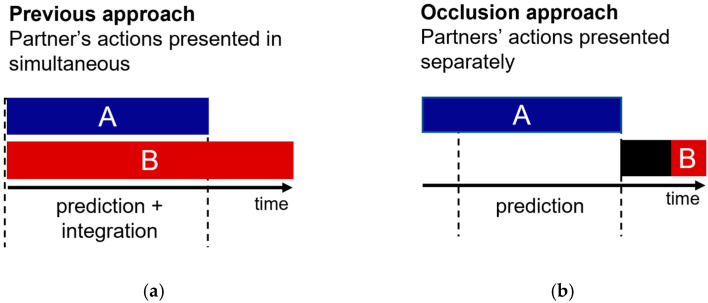
Illustration of the distinction between the experimental approach in previous literature and the occlusion approach used in this study. A and B blocks represent the presentation of each actor. (**a**) In previous studies [3,4,5,6], partners’ actions were presented simultaneously. Although one of the actors was masked, there was still the possibility that observers integrate the cues from both actors rather than proactively predicting one actor from the actions of the other; (**b**) in the current study, we separated the presentation of partner’s actions in time. This allowed us to avoid action integration confounds.

In the last two decades, several studies have concluded that, when processing social interactions, the human visual system relies on predictions about how one partner will react to the other [4,5,6,7,8]. All of these past studies follow the same experimental strategy. Specifically, two interacting point-light-display actors are presented simultaneously (Figure 1a). Critically, one of the actors is easily visible (cue actor), and the other is masked by noise (target actor). Participants must detect the target actor as fast and accurately as possible. Prediction is quantified in the results by faster, or more accurate, visual detection of the target actor when embedded in a meaningful versus non-meaningful interaction (e.g., both actors react to one another versus actors act independently of one another).

A seminal study by Neri and colleagues [6] indicated that participants had less difficulty detecting the target when two actors moved synchronously rather than asynchronously to one another while dancing or fighting. Later, Manera and colleagues [5] extended these observations to the domain of communicative interactions. They found enhanced visual detection of the target actor when both actors behaved congruently (e.g., A asks B to sit down; B sits down) compared to when their actions were incongruent (e.g., A asks B to sit down; B picks something up) or unrelated (e.g., A drinks; B sits down). In addition, visual detection was hindered when the actors’ actions did not match in time [3]. The proposed interpretation for this corpus of evidence is that observing the cue actor triggers predictions about how the target actor will react. In meaningful interactions, these predictions match the actions embedded in the noise and thus facilitate the perception of the target actor. However, the studies above have an interpretation problem: predictions about the target actor occur during and not before the presentation of the target actor. Therefore, it is possible that these studies measured (at least to some extent) the ease of *integration* between the visible actor and the masked actor, rather than measuring the *prediction* of the masked actor based on the actions of its visible partner. 

The importance of considering the role of perceptual integration in social interaction perception is brought to focus by new observations showing that the visual system prioritizes the processing of social interactions as a whole, over the processing of individual actions [9,10,11,12,13,14]. It is widely accepted that the detection of stimuli encompassing multiple parts is facilitated by representing the parts as a whole [15,16]. Therefore, if our visual system processes social configurations as a whole, then the visual detection of interacting partners might be facilitated through perceptual integration. Recent evidence suggests that the visual system treats social scenes in a configurational manner, prioritizing the dyad’s processing over the processing of separate individuals [9,10,11,12,13,14]. Moreover, the observation that Gestalt phenomena are robust to visibility manipulations [17] further supports the idea that perceptual grouping may occur even when part of the stimuli is masked by noise. Hence, an alternative interpretation for previous social prediction findings [4,5,6,7,8] could be delineated as follows: When the point-light displays were spatiotemporally aligned in a meaningful way, their configural patterns tapped into the visual system bias to efficiently bind social information leading to visual detection enhancement. Given this competing account, research directed at understanding the role of prediction in social interaction perception must carefully consider—and control for—a posteriori data integration processes.

The present study investigates the role of prediction in social interaction perception while avoiding the action integration confound identified above. Our hypothesis is in line with past research on this topic: If prediction drives the perception of social interactions, then spatiotemporal simulations of the interaction must be generated in real-time to be compared with the upcoming sensory input. However, in our new test of this old hypothesis, we controlled for action integration confounds by separating the presentation of action and reaction (Figure 1b). 

**Figure 2:**
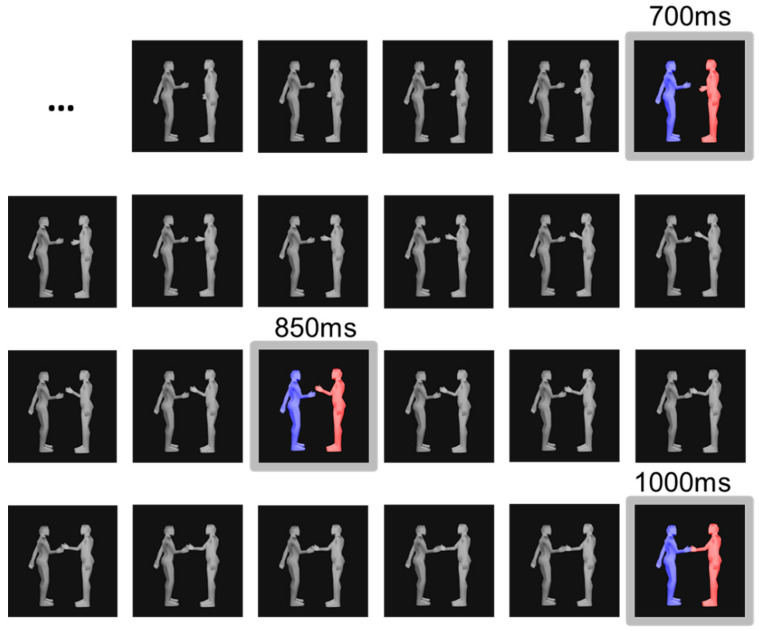
Example of a reaction frame sequence at 60 fps. Coloured frames represent the different still frames at 700 ms, 850 ms and 1000 ms. The grey frames were occluded.

We adapted an occlusion task previously used to study individual action prediction [18]. Participants watched an animated sequence featuring two 3D characters, one red and one blue. Critically, half of the action–reaction sequences were semantically congruent, and the remaining were incongruent. For example, *blue* approaches to shake *red’s* hand, and *red* shake their hand (congruent action–reaction) versus *blue* approaches to shake *red’s* hand, and *red* leans down (incongruent action–reaction). Each sequence starts with an animation of a blue character executing a social action, while a red character is neutral and static. For example, *blue* approaches to shake *red’s* hand, and *red* stands still. Immediately after, instead of presenting the red actor’s reaction (e.g., *red* reciprocally shaking blue’s hand), a black screen covers the animation for a short time (occlusion duration of 700 ms, 850 ms or 1000 ms) until a still frame depicting red’s reaction is shown (reaction frame). The still frame shows *red* at a specific instant of its reaction (Figure 2). In the example of shaking hands, the reaction frame is a still image of *red* at a particular stage of extending its hand, either starting to lift the hand (at 700 ms), mid-way through extending the arm forward (at 850 ms) or grabbing blue’s hand (at 1000 ms). After this, participants judged the congruency of the action–reaction sequences.

Occlusion duration (700 ms, 850 ms, 1000 ms) and reaction frame (at 700 ms, at 850 ms, at 1000 ms) were independently varied to create three temporal alignment conditions: Δ0—the moment depicted in the reaction frame was temporally aligned with the time passed since the start of the occlusion (e.g., 1000 ms were occluded, and the frame showed the reaction after 1000 ms had passed); Δ150—the moment showed in the reaction frame occurred 150 ms before or after the occlusion duration (e.g., 1000 ms were occluded, but the frame depicted the reaction at 850 ms) and Δ300—the moment showed in the reaction frame occurred 300 ms before or after the occlusion duration (e.g., 1000 ms were occluded, but the frame depicted the reaction at 700 ms). 

We predicted that if participants internally simulate the temporal unfolding of the interaction, their congruency judgments will be faster and more accurate when the reaction frame presented after the occlusion corresponds to the time passed since the start of the occlusion. This reasoning is based on the hypothesis that observers internally simulate time-locked expectations of the reactions. Hence, once a frame of the reaction is shown, participants can quickly compare it to their internal representations and determine if it is a congruent or incongruent reaction. Following this reasoning, we expected that performance would deteriorate with increasing distance between the occlusion duration and the moment depicted in the reaction frame (i.e., responses in the Δ0 condition would be faster and more accurate than responses in the Δ150 condition, which would, in turn, be quicker and more accurate than responses in the Δ300 condition). 

Importantly, unlike previous studies, we separated the presentation of the predictive action from the predicted reaction. Our primary goal was to examine action prediction in isolation from action integration. In addition, this study will explore the relationship between individuals’ autistic traits and their sensitivity to temporal deviations in social interactions. A previous study using the simultaneous presentation of two actors in interaction showed that high-functioning-autism (HFA) participants did not automatically use the visible actor’s action to predict the masked partner [4]. Thus, the authors suggest that individuals with HFA show a lower tendency to rely on predictive mechanisms when observing social interactions. However, the experimental approach used in this previous study does not disambiguate between action prediction and action integration. Therefore, it could be that individuals with HFA differently integrate the information from individual actors in a simultaneous interaction. In support of this alternative interpretation, a recent study shows that individuals with higher autistic traits show a lower tendency to process images of two bodies in a social configuration as unitary percept [19]. These observations further motivate using an experimental approach that can disambiguate between predictive and integrative mechanisms.

## 2. Methods

### 2.1. Participants

Twenty participants between 19 and 35 years of age (10 female) were recruited from the University of Birmingham Human Subject Pool. Participants received partial course credit in exchange for one hour of their time. All participants reported normal or corrected-to-normal vision. The University of Birmingham Research Ethics Board approved student participants for credit in this study.

### 2.2. Stimuli

This study used 3D animations of non-verbal dyadic interactions. The animations were created by motion capturing two professional actors. The actors followed scripts designed to generate actions and reactions that could be paired congruently or incongruently. Actions and reactions were recorded separately and then paired to create the congruency conditions. An 11 camera VICON motion capture system (Vicon Motion Systems Ltd., Oxford, UK) was used to capture the movement of two professional actors that followed short scripts. The actors wore 39 reflexive markers on anatomical locations specified in the VICON skeleton template. Motion capture data was recorded at 100 Hz. The data were pre-processed using the VICON Nexus 2.1 software to reconstruct lost trajectories due to marker occlusion. We used Autodesk MotionBuilder software to map the motion capture data onto neutral looking 3D characters. Animations were presented at 60 fps using Psychtoolbox 3 in Matlab 2019a.

Action–reaction animations featured interactions between a *blue* character (action actor) and a *red* actor (reaction actor). For example, *blue* beckons *red* over; *red* takes one step forward. Twenty-four different actions executed by the blue actor follow four interaction themes—offering, helping, greeting and directing. We used two kinematic versions of each action stimuli to avoid repeated presentations of the same animation. For example, there is a basketball and a volleyball version for the action in which blue throws a ball towards red. Each action was paired with two different reactions to create matching congruent versus incongruent sequences (i.e., *blue* throws a ball is paired with *red* jumps upwards in place to catch the ball (congruent) vs. *red* reaches down with their left hand (incongruent). Action–reaction sequences were rendered in left-to-right and right-to-left configurations. There were 96 different sequences (24 actions × 2 reaction congruencies × 2 spatial orientations). See supplementary materials for video examples.

### 2.3. Experimental Design

**Figure 3:**
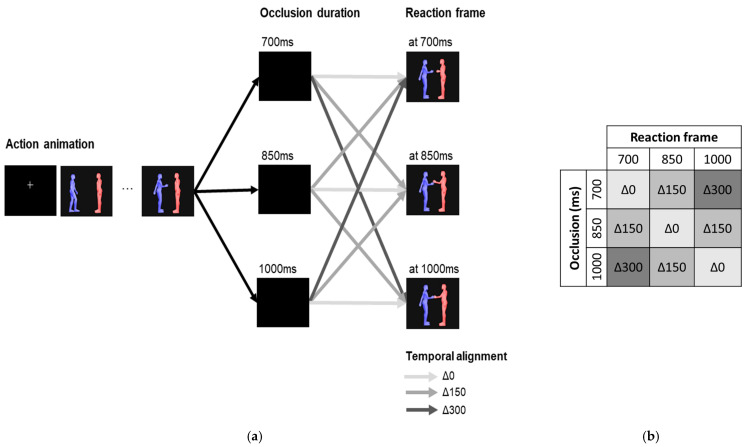
Experimental design. (**a**) The stimuli presentation stages within each trial. Trials began with a 3000 ms animation of a *blue* character performing a social action (e.g., *blue* reaches to shake *red’s* hand). A black occlusion frame then blocked the animation. Following this, a frame representing a specific instant of the *red* character’s reaction was shown (e.g., *red* reaches its hand forward). (**b**) The three occlusion durations (700, 850, 1000 ms) were independently combined with the three different reaction frames (at 700, at 850, at 1000 ms) to generate the temporal alignment conditions: Δ0—the moment depicted in the reaction frame is temporally aligned with the duration of the occlusion; Δ150—the moment showed in the reaction frame occurs 150 ms before or after the occlusion duration; Δ300—the moment depicted in the reaction frame occurs 300 ms before or after the occlusion duration.

The three occlusion durations (700, 850 and 1000 ms) were independently combined with three reaction frames (at 700, at 850 and at 1000 ms). Figure 3b illustrates the resulting nine occlusion-frame combinations. These occlusion-frame combinations give rise to the following condition distribution: 3/9 occlusion-frame combinations correspond to the Δ0 temporal alignment condition; 4/9 occlusion-frame combinations correspond to the Δ150 temporal alignment condition; 2/9 occlusion-frame combinations correspond to the Δ300 temporal alignment condition. We randomly assigned the 96 animations to the different occlusion-frame combinations to closely follow the resulting condition distribution. This led to 36 Δ0 trials, 40 Δ150 trials and 20 Δ300 trials.

The 96 trials were presented in 6 blocks. Each block had 16 trials. Four distinct *blue* actions were paired with 2 different *red* reactions to generate congruent and incongruent action sequences that started with the same action (e.g., *congruent sequence*: ‘Asks for help finding a lost object on the floor’/”Reaches down with the left hand’ vs. *incongruent sequence*: ‘Asks for help finding a lost object on the floor’/”Jumps upwards in place’). Action sequences were presented in left–right and right–left spatial arrangements. The *blue* actions within each block appeared paired with both congruent and incongruent *red* reactions an equal number of times. Thus, blocks were fully balanced for congruent and incongruent action–reaction pairs. Table A1, Table A2, Table A3, Table A4, Table A5 and Table A6 in Appendix A describes the action–reaction pairs within each block. Blocks were presented in random order, and trials within each block were randomized. Importantly, across the whole experiment, congruency was balanced within each temporal alignment condition. 

### 2.4. Procedure

Before starting, participants were informed that the experiment was divided into six blocks. The experimenter explained that each block featured several animations of social interactions between a *blue* actor and a *red* actor. At the start of each animation, the blue actor would execute a social action, to which the *red* actor would react. Participants were informed that their task would be to judge whether *red’s* reaction corresponded to a congruent continuation of the interaction. The experimenter explained that in congruent trials, the action of the *blue* actor and the reaction of the *red* actor belonged to the same social event and would be related. The experimenter offered a verbal example of the type of interactions the participant would see. For instance, in a congruent trial, one of the actors might point upwards to direct the other’s attention, and the second actor will react straightforwardly by looking upwards. In an incongruent sequence, the second actor would perform an unrelated action. For example, one of the actors would point upwards to direct the other’s attention, and the second actor would step forward. Participants were told that each block would feature four *blue* actions and two *red* reactions. The experimenter explained that each block had a familiarization and testing stage. A familiarization stage was completed at the start of each block to ensure that the familiarization effects were constant across all blocks. During the familiarization stage, participants observed four blue actions and two red reactions presented separately and accompanied by a descriptive sentence (e.g., *blue* hands over a small object). Participants attended to the two versions of each action, and both actions and reactions were presented in the left and right spatial locations. The experimenter highlighted that although the blue and red actions appeared separately during familiarization, they would later appear paired as action–reaction sequences in the testing stage.

During the testing stage, each trial began with a fixation screen (1000–1500 ms) followed by a *blue* action animation (3000 ms) presented at 60 fps, after which an occlusion frame was presented for either 700, 850 or 1000 ms. Subsequentially, a static frame of *red*’s reaction appeared on the screen—this frame corresponded to a still of red at 700 ms, 850 ms or 1000 ms into the reaction. Participants were instructed to indicate, as fast and accurately as possible, whether *red’s* reaction frame was or not a congruent continuation to the interaction initiated by the *blue* character. Participants used the *Q* and *p* keys to indicate their responses. Key-response association was counterbalanced within participants. The reaction frame stayed on the screen until response input. Participants responded using the keyboard and were asked to keep their index fingers over the keys throughout the experiment to ensure accurate reaction times. 

On completion of the session, participants filled in the Autism-Spectrum Quotient questionnaire (AQ) [20]. The AQ questionnaire consists of 50 items grouped into five subscales: communication (e.g., Other people frequently tell me that what I’ve said is impolite, even though I think it is polite); social skill (e.g., I prefer to do things with others rather than on my own); attention switching (e.g., I prefer to do things the same way over and over again); attention to detail (e.g., I often notice small sounds when others do not); imagination (e.g., If I try to imagine something, I find it very easy to create a picture in my mind). AQ scores indicate the degree to which an individual reveals autistic traits. AQ scores range from 0 to 50, with higher scores corresponding to a larger prevalence of autistic traits. It is important to caution that this scale is not used alone in clinical diagnosis. However, an AQ score of 32 is suggested by Baron-Cohen and colleagues [20] to be a useful cut-off for distinguishing individuals with clinical levels of autistic traits. The full experimental session had a duration of 60 min. 

## 3. Analysis

The analysis tested whether the temporal alignment between the time passed since the end of the action prompt (occlusion duration) and the static frame representing the unfolding of the reaction (reaction frame) interfered with participants’ ability to categorize the congruency of the observed interactions. The dependent measures were accuracy and reaction time. Overall, accuracy was 77% (SD = 9.6), and the average reaction time was 1.05 s (SD = 0.27). Responses to the interaction sequences showed a marked speed-accuracy tradeoff revealed by a strong negative correlation between accuracy and reaction times, *r* (22) = −82, *p* < 0.0001, 95% CI [−0.91, −0.62]. Given that accuracy and reaction time were strongly correlated, we computed a composite score that combines both measures to best capture participants’ responses. Linear integrated speed-accuracy scores (LISAS) were calculated per subject × condition using the equation $LISAS_{\;j}=\bar{RT_{j}}+\frac{S_{RT_{j}}}{S_{PE_{j}}}\times PE_{j}$ where $\bar{RT_{j}}$ is the participant mean RT on correct-response trials in conditions j, and $PE_{j}$ is the participant proportion of errors in condition j. $S_{RT}{}_{j}$ and $S_{PE}{}_{j}$ are the across-trial sample standard deviations of the participant in condition j. Both the mean $\bar{RT_{j}}$ and $S_{RT}{}_{j}$ pertain to correct trials [21,22,23]. This quantification puts unequal weights on speed and accuracy depending on the accuracy level. Note that the weighted proportion of errors is added to reaction times. Thus, lower LISAS correspond to higher task performance. 

## 4. Results

**Figure 4:**
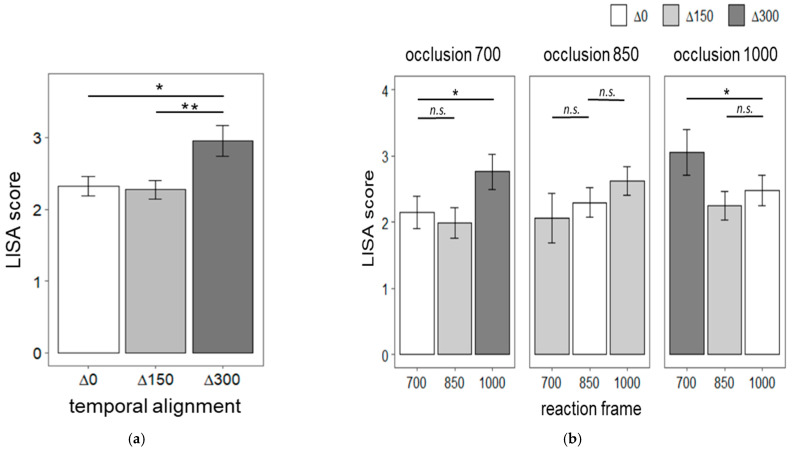
Mean Linear Speed-Accuracy Scores (LISAS) of observers’ congruency responses. LISAS are a compositive score of reaction time and accuracy. In this measurement, reaction times are weighted by the proportion of errors. Thus, lower LISAS indicate higher task performance. (**a**) LISAS for the temporal alignment conditions: occlusion duration and reaction frame matched (Δ0), 150 ms deviations (Δ150), or 300 ms deviations (Δ300). (**b**) Mean Linear Speed-Accuracy Scores (LISAS) of observers’ congruency responses at all combinations of occlusion duration (700, 850, 1000 ms) and reaction frame (700, 850, 1000 ms). Error bars are +/− standard error of the mean; n.s. indicates a non-significant comparison; * indicates a comparison with *p* < 0.05; ** indicates a comparison with *p* < 0.01.

Figure 4a shows the linear speed-accuracy scores (LISAS) for Δ0, Δ150 and Δ300 temporal alignment conditions. A repeated-measures ANOVA indicated that the distance between occlusion duration and reaction frame timing interferes with speed-accuracy scores, F (2,38) = 11.01, *p* < 0.0001, η^2^ = 0.09. Post-hoc pairwise t-tests with Bonferroni correction showed that both Δ0 (mean = 2.33, SD = 0.84) and Δ150 (mean =2.27, SD = 0.82) had lower speed-accuracy scores than Δ300 (mean = 2.95, SD = 1.27), *t* (19) = −3.73, *p* < 0.01, 95% CI [1.93 2.72] and *t* (19) = −3.34, *p* < 0.05, 95% CI [1.89, 2.66], respectively. Δ0 and Δ150 did not significantly differ. Next, we tested if speed-accuracy scores within each occlusion duration level (Figure 4b). Specifically, we independently tested for each occlusion level if the LISAS in the Δ0 temporal alignment condition (i.e., oclusion700-frame700, oclusion850-frame850 and oclusin1000-frame1000) were lower than the LISAS corresponding to temporal deviations. For example, for the occlusion duration of 700 ms, LISAS for oclusion700-frame700 trials were lower than oclusion700-frame850 and oclusion700-frame1000 LISAS. Planned contrasts revealed that LISAS for 700 ms occlusion followed by a reaction frame at 700 ms were lower than when a reaction frame at 1000 ms followed the same occlusion duration, *t* (19) = 2.14, *p <* 0.05. Additionally, LISAS for 1000 ms occlusion followed by a reaction frame at 1000 ms were lower than when a reaction frame at 700 ms followed the same occlusion duration, *t* (19) = 2.13, *p* < 0.05. The remaining comparisons were not significant.

**Figure 5:**
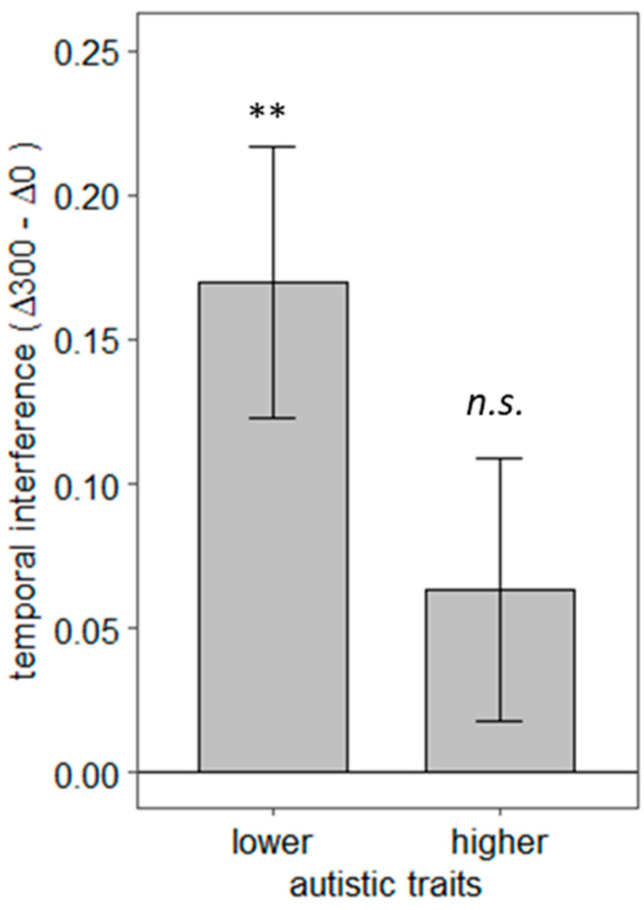
Link between autistic traits and sensitivity to temporal deviations in observed social interactions. Y-axis shows the mean temporal interference index that corresponds to the difference between LISAS at Δ300 and Δ0. Positive values that significantly differ from zero indicate sensitivity to temporal deviations. Participants were split into higher and lower autistic trait groups according to their autism quotient score, shown in the x-axis. Error bars represent +/− one standard error of the mean; n.s. indicates a non-significant comparison; ** indicates a comparison with *p* < 0.01.

To explore the link between autistic traits and individual differences in social interaction prediction, we divided our participant sample into two groups using a median split on the autistic quotient. The mean AQ score was 18.55 (SD =7.7). A median split meant that 8 participants scoring between 11 and 16 were considered in the lower autistic traits group, and 12 participants scoring between 17 and 44 were assigned to the higher autistic traits group. A temporal interference score was computed for each participant by calculating the difference between LISAS at Δ300 and LISAS at Δ0. This score allowed us to index participants’ sensitivity to the temporal alignment of the action–reaction sequences. If the difference between LISAS at Δ300 and Δ0 is statistically greater than zero, we can conclude that temporal deviations hindered participants’ congruency judgments. Figure 5 shows the mean interference scores for lower and higher autistic traits groups. Bonferroni corrected one-tailed t-tests revealed that the lower autistic trait group was affected by the temporal deviation of 300 ms, *t* (7) = 3.62, *p* < 0.01, 95% CI [−∞, 2.66], *d* = 1.28. *In contrast*, the higher autistic trait group was not, *t* (11) = 1.40, *p* = 0.10, *d* = 0.40. A Welch two-sample t-test showed that lower and higher autistic groups did not differ significantly in temporal interference, *t* (16.82) = 1.63, *p =* 0.12, *d* = 0.73. To further explore the link between autistic traits and social interaction prediction, we performed a Pearson correlation test between participants AQ scores and their temporal interference index. This test showed a non-significant trend toward a positive link between temporal interference and AQ scores, *r* (18) = −0.30, *p* = 0.19. Post-hoc power analyses were conducted. Using eight participants (lower autistic group), the one-sample t-test would be sensitive to an effect size of *d* = 0.5 with 37% power. Using 12 participants (higher autistic group), the power of this test is 36%. The Welch two-sample t-test comparing both autistic trait groups would be sensitive to an effect size of *d* = 0.5 with only 17% power using the sample sizes in our study. Using 20 participants, a Pearson correlation test would be sensitive to an r value of 0.3 with only 25% power. These post-hoc power analyses show that our study does not have enough power to reliably capture the relationship between participants’ autistic traits and their sensitivity to temporal deviations.

## 5. Discussion

The current study isolated *action prediction*—a priori representations of an upcoming reaction to an observed action—from *action integration*—a posteriori processing of simultaneous actions between interacting partners. Our main finding is that judging the congruency of an observed action–reaction sequence was facilitated when the observed reaction was temporally aligned with expectations about its unfolding, compared to when it deviated by 300 ms. Because performance in judging the congruence of the action–reaction sequences depended on temporal alignment, we can conclude that the findings reflect the effects of time-based action predictions rather than a posteriori action integration. Our study thus provides additional evidence supporting the notion that human perceptual systems generate time-locked action predictions about observed social interactions [1,2]. 

Interestingly, while deviations of 300 ms affected most observers’ ability to judge the social interactions, deviations of 150 ms did not show any interference. This observation suggests that temporal resolution in predictive representations is above 150 ms. From a computational point of view, reducing the level of detail of internal representations is a viable strategy to optimize predictive mechanisms. As a result, predictive systems may become more robust to external uncertainty by relaxing their sensitivity to deviations between sensory input and internal predictive representations. However, this strategy comes at the cost of information loss [24]. We suggest that, in our study, the trade-off point between robust and precise temporal predictions was somewhere between 150 ms and 300 ms. In future research, a parametric manipulation of the temporal deviations in the experimental task can probe this resolution.

Whereas the results from the present study show the involvement of time-based predictive processes in the perception of social interactions, it is essential to acknowledge that it is not possible to conclude if this link is specific to social interactions. Real-time predictions are known to be engaged in the perception of individuals in isolation [18] and extend to non-biological motion [25]. Moreover, it is essential to recognize that the present study cannot offer insight into whether action–reaction predictions are purely perceptual or engage the motor system. In contrast to the motor theories that dominated social cognition in the early 2000s [26], recent neuroscientific evidence supports a purely perceptual account of social interaction perception. Specifically, several recent studies independently reported that the superior temporal sulcus (STS) is selectively involved in the perception of social interactions [27,28,29]. The STS is a visual area known for its engagement in the visual processing of biological motion. However, long-onset latencies in STS cells have been reported, and these could, in theory, allow for the integration of top-down input from pre-motor areas [30]. Future research is needed to probe if the STS works in tandem with pre-motor areas to formulate time-based interaction predictions.

Furthermore, the current study offers preliminary insights on the link between social predictions and autistic traits. Specifically, we observed that participants with higher autistic traits (i.e., non-clinical propensity to empathize less strongly with others and engage in systemized thinking) are insensitive to temporal deviations of 300 ms when judging the coherence of social interactions. This preliminary observation aligns well with the notion that Autism Spectrum Disorder (ASD) is rooted in disordered predictive mechanisms [31]. According to this proposition, a decreased ability to recognize predictive links between environmental events may explain the diverse behavioural traits in ASD. We caution against any conclusive interpretations of these findings due to lack of power in our study. Nonetheless, our observations may motivate and inform the design of future studies that are directly aimed at capturing individual differences in social interaction perception. Based on the observed effect sizes in our data, we suggest that future studies should include at least 59 participants in each of the lower and higher autistic traits groups to achieve a power of 85%. The AQ score threshold for the inclusion of participants in lower and higher autistic trait groups should be defined a priori. 

The contribution of the present study to our current understanding of social interaction perception is two-fold. First, the study offers new behavioural evidence supporting that prediction underlies social interaction perception. In addition, the study puts forward a new methodological approach to avoid action integration confounds when measuring social interaction prediction. The observation of prediction independently of simultaneous action integration does not preclude the notion that the priors in the predictive process —i.e., the long-term semantic knowledge about social interactions—are encoded in an integrative fashion [10,32,33]. Integrative priors offer a reasonable explanation to the observed facilitation in detecting a congruent partner when observing social interactions [4,5,6,7,8]. In line with this reasoning, our study advances an experimental approach to isolate the contribution of integrative priors (a priori expectations about social interactions) from the contribution of online action integration processing to the perception of social interactions. We hope that the current research will stimulate further experimental developments to disentangle the interdependencies between action prediction and action integration mechanisms and, in this way, advance the understanding of how humans perceive dynamic social interactions.

## Data Availability

The data presented in this study are available in the Supplementary Materials.

## Supplementary Materials

The following are available online at https://www.mdpi.com/article/10.3390/brainsci12040432/s1. Video_S1.mp4: Example of congruent interaction—Points downward to direct someone’s attention. Leans forward looking down.; Video_S2.mp4: Example of incongruent interaction—Points downward to direct someone’s attention. Reaches forward with the right hand.; Video_S3.mp4: Example of congruent interaction—Opens a door. Takes one step forward.; Video_S4.mp4: Example of incongruent interaction—Opens a door. Reaches down with the left hand.; Video_S5.mp4: Example of congruent interaction—Passes over a large object. Opens both arms forward; Video_S6.mp4: Example of incongruent interaction—Passes over a large object. Leans forward looking down; Data.csv: Experimental data.

## Appendix A

**Table A1:** Action–reaction sequences in Block A.

	Reaction (Red Actor)	
Action (Blue Actor)	Congruent	Incongruent
Bends over in pain, looking for comfort	Reaches forward with the right hand	Leans forward while looking down
Extends hand for a handshake	Reaches forward with the right hand	Leans forward while looking down
Throws a ball towards someone’s feet	Leans forward while looking down	Reaches forward with the right hand
Points to direct someone’s attention	Leans forward while looking down	Reaches forward with the right hand

**Table A2:** Action–reaction sequences in Block B.

	Reaction (Red Actor)	
Action (Blue Actor)	Congruent	Incongruent
Reaches over for a hug	Opens both arms forward	Reaches forward with the right hand
Picks up an object and hands it over	Reaches forward with the right hand	Opens both arms forward
Expresses sadness	Opens both arms forward	Reaches forward with the right hand
Asks someone to indicate a choice betweentwo options	Reaches forward with the right hand	Opens both arms forward

**Table A3:** Action–reaction sequences in Block C.

	Reaction (Red Actor)	
Action (Blue Actor)	Congruent	Incongruent
Asks for help finding a lost object on the floor	Reaches down with the left hand	Jumps upwards in place
Throws over a ball	Jumps upwards in place	Reaches down with the left hand
Approaches to greet someone with a hi-5	Jumps upwards in place	Reaches down with the left hand
Is seated on the floor and asks for help getting up	Reaches down with the left hand	Jumps upwards in place

**Table A4:** Action–reaction sequences in Block D.

	Reaction (Red Actor)	
Action (Blue Actor)	Congruent	Incongruent
Beckons someone over	Takes one step forward	Jumps upwards in place
Asks for help reaching something stored on a high location	Jumps upwards in place	Takes one step forward
Extends arm to offer an item	Takes one step forward	Jumps upwards in place
Convinces someone else to jump up	Jumps upwards in place	Takes one step forward

**Table A5:** Action–reaction sequences in Block E.

	Reaction (Red Actor)	
Action (Blue Actor)	Congruent	Incongruent
Asks someone to accept a gift	Opens both arms forward	Leans forward while looking down
Signals the need to see behind someone else	Leans forward while looking down	Opens both arms forward
Passes over a large object	Opens both arms forward	Leans forward while looking down
Bows over with respect	Leans forward while looking down	Opens both arms forward

**Table A6:** Action–reaction sequences in Block F.

	Reaction (Red Actor)	
Action (Blue Actor)	Congruent	Incongruent
Proposes by kneeling and offering a ring	Reaches down with the left hand	Takes one step forward
Performs a royal curtsy	Reaches down with the left hand	Takes one step forward
Expresses needing help	Takes one step forward	Reaches down with the left hand
Opens a door	Takes one step forward	Reaches down with the left hand

Note. Table specifying the content of the action–reaction animations comprised in each of the six experimental blocks. The animations followed four themes—offering, helping, greeting and directing. In addition, there were six possible reactions —walks over, reaches ahead, reaches down, opens arms, looks down and jumps. Interaction themes were balanced across the possible reactions to avoid semantic confounds.
